# Multiple Independent Gene Disorders Causing Bardet–Biedl Syndrome, Congenital Hypothyroidism, and Hearing Loss in a Single Indian Patient

**DOI:** 10.3390/brainsci13081210

**Published:** 2023-08-16

**Authors:** Isabella Peixoto de Barcelos, Dong Li, Deborah Watson, Elizabeth M. McCormick, Lisa Elden, Thomas S. Aleman, Erin C. O’Neil, Marni J. Falk, Hakon Hakonarson

**Affiliations:** 1 Center for Applied Genomics, Division of Human Genetics, Department of Pediatrics, Children’s Hospital of Philadelphia, Philadelphia, PA 19104, USA; barcelosi@email.chop.edu (I.P.d.B.); lid2@chop.edu (D.L.);; 2 Mitochondrial Medicine Frontier Program, Division of Human Genetics, Department of Pediatrics, Children’s Hospital of Philadelphia, Philadelphia, PA 19104, USA; mccormicke@chop.edu (E.M.M.); falkm@chop.edu (M.J.F.); 3 Division of Otolaryngology, Perelman School of Medicine, University of Pennsylvania, Philadelphia, PA 19104, USA; elden@chop.edu; 4 Division of Ophthalmology, Children’s Hospital of Philadelphia, Philadelphia, PA 19104, USA; aleman@pennmedicine.upenn.edu (T.S.A.); oneile@chop.edu (E.C.O.); 5 Center for Advanced Retinal and Ocular Therapeutics (CAROT), Department of Ophthalmology, University of Pennsylvania Perelman School of Medicine, Philadelphia, PA 19104, USA; 6 Scheie Eye Institute at the Perelman Center for Advanced Medicine, Department of Ophthalmology, University of Pennsylvania, Philadelphia, PA 19104, USA; 7 Department of Pediatrics, Perelman School of Medicine, University of Pennsylvania, Philadelphia, PA 19104, USA

**Keywords:** *BBS6*, STR, *DUOX2*, *TNNT2*, consanguinity

## Abstract

We report a 20-year-old, female, adopted Indian patient with over 662 Mb regions of homozy-gosity who presented with intellectual disability, ataxia, schizophrenia, retinal dystrophy, moder-ate-to-severe progressive sensorineural hearing loss (SNHL), congenital hypothyroidism, cleft mi-tral valve with mild mitral valve regurgitation, and dysmorphic features. Exome analysis first on a clinical basis and subsequently on research reanalysis uncovered pathogenic variants in three nu-clear genes following two modes of inheritance that were causal to her complex phenotype. These included (1) compound heterozygous variants in *BBS6* potentially causative for Bardet–Biedl syn-drome 6; (2) a homozygous, known pathogenic variant in the stereocilin (*STRC*) gene associated with nonsyndromic deafness; and (3) a homozygous variant in dual oxidase 2 (*DUOX2*) gene asso-ciated with congenital hypothyroidism. A variant of uncertain significance was identified in a fourth gene, troponin T2 (*TNNT2*), associated with cardiomyopathy but not the cleft mitral valve, with mild mitral regurgitation seen in this case. This patient was the product of an apparent first-degree relationship, explaining the multiple independent inherited findings. This case high-lights the need to carefully evaluate multiple independent genetic etiologies for complex pheno-types, particularly in the case of consanguinity, rather than presuming unexplained features are expansions of known gene disorders.

## 1. Introduction

Identifying multiple genetic diseases co-occurring in the same patient can occur in approximately 5% of complex phenotypes and increases in frequency in cases with high degrees of consanguinity [1]. Here, we report a highly consanguineous Indian adopted girl who presented with a highly complex phenotype involving multiple congenital anomalies and developed progressive multisystem involvement over time. Genome-wide SNP array analysis confirmed extensive regions of homozygosity by descent consistent with suspected first-degree consanguinity. Exome analysis of the proband conducted on a clinical basis, and complemented by research reanalysis, identified three nuclear genes harboring homozygous variants and a fourth gene surprisingly having compound heterozygous variants, that collectively caused her overall clinical manifestations with classical features of Bardet–Biedl syndrome, congenital deafness, congenital hypothyroidism, and cleft mitral valve. Specifically, the proband had Bardet–Biedel syndrome type 6 (*BBS6*) caused by compound heterozygous variants in *BBS6* (also known as MKKS) that manifests as postaxial polydactyly, truncal obesity, retinitis pigmentosa, kidney defects, and intellectual disability due to dysfunction of the centrosome chaperonin (OMIM # 605231). Her congenital hearing loss resulted from *STRC* childhood-onset deafness associated with a homozygous pathogenic variant in the stereocilin gene (OMIM # 603720). Her congenital hypothyroidism resulted from thyroid dyshormonogenesis type 6 caused by homozygous pathogenic variants in dual oxidase 2 (*DUOX2*) (OMIM # 607200). Finally, there was not enough evidence to associate her cleft mitral valve and mild mitral regurgitation with an autosomal dominant variant of uncertain significance in troponin T, *TNNT2* (OMIM # 191045), as this gene has only previously been associated with cardiomyopathy, which has not been seen in our case to date. Her extensive homozygosity by descent stemming from a consanguineous union was likely related to her inheriting two homozygous recessive disorders (*STRC* and *DUOX2*). Her complete clinical disorder resulted from a third gene disorder (*BBS6*), resulting from compound heterozygous variants.

## 2. Case Report

### 2.1. Clinical Presentation

We report a 20-year-old Indian girl adopted at three months of age from an orphanage where she had unknown parentage from a suspected first-degree consanguineous union. Informed consent was obtained from her adoptive parents per The Children's Hospital of Philadelphia IRB protocol #IRB 06-004886 for research, including photos for publication in January 2022.

Congenital anomalies: She was diagnosed with four-extremity postaxial polydactyly at birth, some with bony remnants. At age four months, congenital hypothyroidism was diagnosed. Dysmorphic facial features were present, including thinning hair, synophrys, bitemporal narrowing, thick eyebrows, broad nasal bridge, hirsutism of the upper lip, thin upper lip, and an increased gap between the upper central incisors. Additionally, she had inverted nipples, a left fifth proximal interphalangeal (PIP) joint contracture fixed to slightly less than 90 degrees, a wide 1/2 toe sandal gap bilaterally, and sharp contours in her distal palm > sole radial border following remote polydactyly repair.

**Figure 1:**
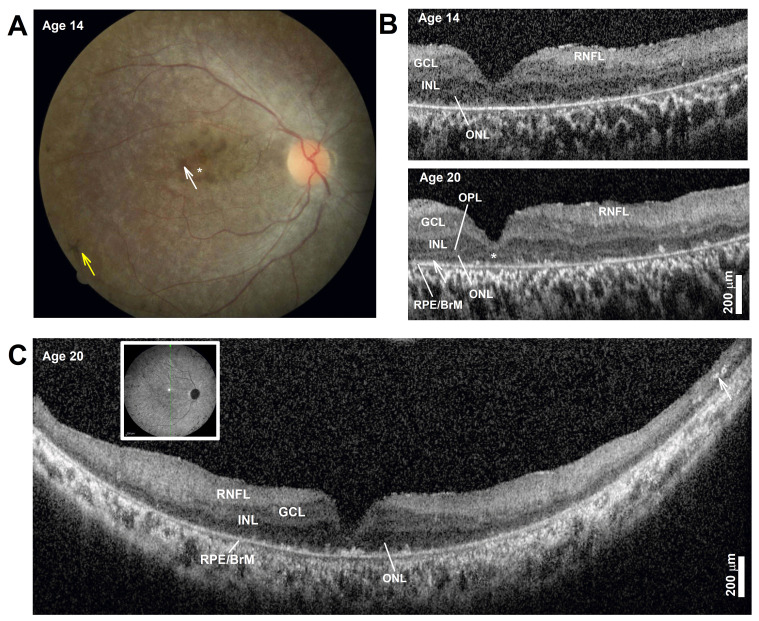
(**A**) Color fundus photography of the central retina. The asterisk marks depigmented foveal center, the white arrow points to parafoveal islands of better-preserved retinal pigment epithelium (RPE), and the yellow arrow points to bone spicule pigment. (**B**) Horizontal, 3 mm, SD-OCT cross-section through the fovea and nasal retina at two ages. Nuclear layers are labeled: outer nuclear layer = ONL, inner nuclear layer = INL, ganglion cell layer = GCL, retinal nerve fiber layer = RNFL, and basal retinal pigment epithelium = RPE. The ONL can be seen bracketed between the outer plexiform layer (OPL) and the RPE/Bruch’s membrane (RPE/BrM). Arrow in the parafovea at age 20 points to a segment where signals superficial to the RPE may correspond with detectable but short inner and/or outer segments. (**C**) Horizontal, 16 mm, SD-OCT vertical cross-section through the fovea at age 20. Inset shows the location of the scan. Arrow points to intraretinal hyperreflectivity corresponding to intraretinal pigment migration and bone spicule formation in a thinner mid-peripheral retina. Scale bars to the bottom right. Only the right eye is shown for clarity; left eye is nearly identical.

Ophthalmologic findings: During infancy, she developed nystagmus. Later visual symptoms included difficulties seeing at night and in dim or brightly lit places around age 6. Examination from presentation to the clinic at age 8 to age 20 showed best-corrected visual acuities ranged from 20/200–20/400 in each eye. Her full-field electroretinogram (ERG) was nondetectable for either rod- or cone-mediated responses. A fundus exam documented at age 14 showed a normal optic nerve. There was an overall mottled depigmentation of the RPE within the central retina with small islands of better-preserved appearance in the parafovea (Figure 1A, white arrow) surrounded by a depigmented foveal center (Figure 1A, asterisk). Bone spicule pigmentation was present in the near midperiphery (Figure 1A, yellow arrow). Spectral domain optical coherence tomography (OCT) imaging showed severe foveal thinning with segments of a thin photoreceptor outer nuclear layer (ONL) only visible in the parafovea and at the foveal center (Figure 1B). The inner segment ellipsoid segment (EZ) and outer segment interdigitation signals (IZ) were undetectable except in small segments in the parafovea (Figure 1B, OCT, arrow). The lamination changed very little over the ensuing years from age 14 to her last exam at age 20, except for foveal thinning and thickening of the inner retinal laminae that corresponded with the RNFL, a consequence of remodeling (Figure 1B) [2]. Long, 16 mm, vertical SD-OCT scans into the midperiphery at age 20 redemonstrated a detectable but thin photoreceptor ONL (Figure 1C). A more peripheral retina becomes bilaminar, with a thick superficial “RNFL” and a deeper hyporeflective band continuous to the central inner nuclear layer (INL) band. At the end of the scans, there was overall thinning and the appearance of hyperreflective lesions that correspond to intraretinal pigment migration, which corresponds with bone spicule pigmentation on fundus exam. The pattern corresponds to an early-onset and severe inherited retinal degeneration (EORD) or juvenile retinitis pigmentosa (RP) with a peculiar predilection for early foveal abnormalities causing severe central vision loss and abnormal visual acuities. Her most recent best-corrected visual acuity at the age of 20 years was without correction 20/300 right; 25/500 left; 20/250 both, and with correction 20/300 right; 20/400 left; and 20/200 both eyes. She is legally blind and has nyctalopia, and continues to learn Braille. She received education through a school for blind students, uses a cane, and reads in Braille and large printed letters.

**Figure 2:**
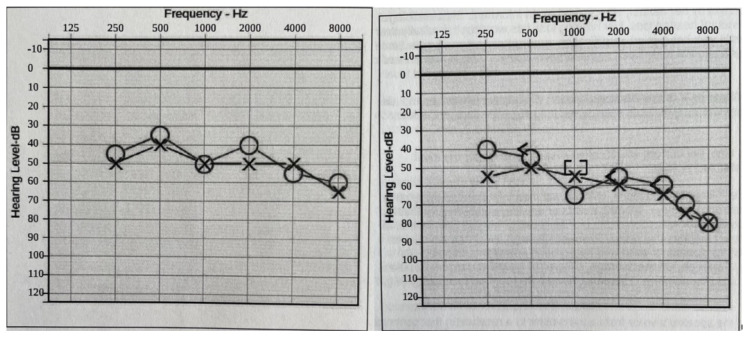
Audiograms (**Left**: at 16 years old. **Right**: at 18 years old). Bilateral moderate-to-profound progressive hearing loss. The sign “O” stands for right ear unmasked air conduction. The symbol “X” stands for left ear unmasked air conduction. The symbol “[” stands for right ear masked bone conduction. The sign “]” stands for right ear masked bone conduction.

Hearing loss: At two years of age, she was diagnosed with baseline moderate sensorineural hearing loss (SNHL) with an additional conductive component due to recurrent otitis media (requiring repeat pressure equalizer tube placement). An MRI of the temporal bone showed normal inner ear and auditory nerve anatomy. As she grew older, the conductive component of her hearing loss resolved as her middle ear function improved. Still, she had a slow progression of the SNHL, such that at age 20, her hearing is classified as moderate-to-severe SNHL, and she needs bilateral hearing aids to improve functioning (Figure 2).

Cardiac problems: A cleft mitral valve (3 mm gap) with mild mitral valve regurgitation was found; the findings remained mild and stable, and the patient is asymptomatic. Apparent cardiomegaly with biventricular enlargement was described on chest CT in 2011, although no evidence of cardiomyopathy was seen on subsequent echocardiograms, which were last performed when the patient was 18 years old.

Neurodevelopmental disabilities and mood disorder: She was diagnosed with developmental delay and hypotonia. She began walking at 1.5 years and was not toilet-trained until age 8. Her speech was significantly delayed, speaking single words by age 3 and two-word sentences by age 4–5. Around 15 years, her Wechsler scale score was 45 (although caution was noted due to her hearing and vision loss), with overall function scoring in the moderate range of intellectual disability. As a toddler, she started to have mood swings in which she became aggressive toward herself and others. At 16 years of age, she developed psychiatric symptoms with episodes of hallucinations and was talking to imaginary friends (destructively and negatively), leading to a diagnosis and treatment for schizophrenia; her schizophrenia has responded well to antipsychotic medication. Her neurologic exam at age 18 indicated slow response times to questions, diminished visual acuity, nystagmus in central gaze, mild hypotonia, dysarthria, wide-based gait, and difficulty with coordination, balance, and dysmetria. The last neuropsychological evaluation, considering her hearing and vision loss deficiencies, evidenced overall intellectual function in the moderate ID range.

Endocrine problems: In addition to congenital hypothyroidism, she experienced precocious puberty managed with medication. Brain MRI showed a smaller-than-expected pituitary gland (measuring less than 3 mm in craniocaudal diameter). She had dyslipidemia, with a history of a two-fold increase in triglycerides that improved to only slightly elevated at age 18 years. Her previously elevated HbA1C in the prediabetes range (5.9% at 14 years) normalized (5.2% by age 18) in association with her diet and exercise plan. Her congenital hypothyroidism is stable on Synthroid.

Skeletal problems: Skeletal findings include S-shaped scoliotic mild scoliosis convex to the right in the thoracic and convex to the left in the lumbar without surgical intervention. A mild increase in lumbar lordosis is present. She also wears ankle–foot orthotics to improve her balance while walking.

**Figure 3:**
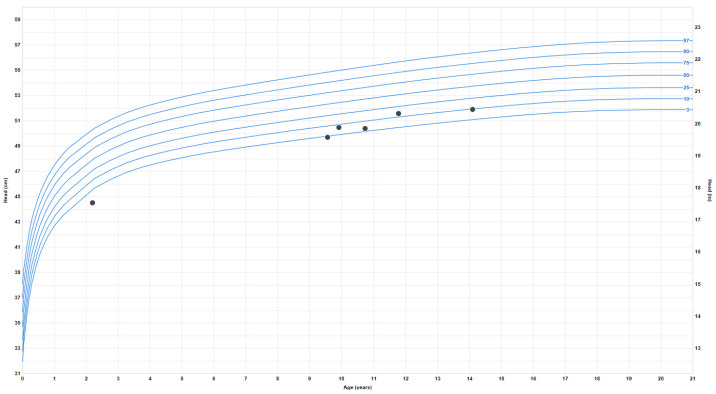
Head circumference or occipitofrontal circumference (OFC) measurements.

Growth problems: She experienced early failure to thrive, which resolved during late childhood when she began to have relative overweight with truncal obesity. Her height started to plateau around age 13–14, and her final adult height is around the 50th percentile. At age 9, the weight progressively increased from the 4th percentile to the 80th percentile and has plateaued since age 16. Her BMI also increased around ten years old from the 7th percentile to the 85th percentile (at 20 years). The earliest head circumference measurement available was at 8 months, in the second percentile, and the last measurement at age 18 was 52.5 cm (fourth percentile) (Figure 3).

Gastrointestinal problems: Chronic diarrhea was present from infancy until age 4–5, later diagnosed as lactose intolerance.

Lymphatic problems: During early childhood, she presented with several enlarged lymph nodes (left submandibular, left supraclavicular, and left axillary lymph nodes) exhibiting homogeneous enhancement without cavitation or surrounding inflammatory changes, as well as enlarged axillary lymph nodes without malignancy or infectious disease; these findings are not present anymore.

### 2.2. Genetic Testing Results

Genome-wide single-nucleotide polymorphism (SNP) microarray analysis was clinically performed, with no pathogenic deletions or duplications detected. However, multiple large (>10 megabases, Mb) regions of homozygosity were distributed across the entire genome. These regions of homozygosity totaled over 662 Mb and represented 22% of her genome, consistent with her being the offspring of a union between first-degree relatives [3]. However, multiple generations of inbreeding cannot be excluded in this case.

The whole-exome sequencing was remarkable for pathogenic or suspected pathogenic variants being identified in three different nuclear genes and one VUS without clinical correspondence to date (see Table 1). While segregation analysis was impossible due to their inability to identify or evaluate her biological parents, these genes provide plausible etiologies for her complex clinical presentation. The specific clinical syndromes and associate genes include the following:

1. Bardet–Biedl syndrome 6 (OMIM: 605231) due to presumptive compound heterozygous variants in *BBS6* identified in a gene panel and confirmed by exome analysis (not in one of the regions of homozygosity). The diagnosis of BBS is consistent with her clinical features of four-extremity polydactyly, truncal obesity, rod-cone retinal dystrophy involving severely decreased visual acuity, chronic otitis media requiring repeat PE tube placement, developmental delay, behavioral issues, and learning/intellectual disability. She does not have other features typical of BBS, such as renal/pelvic anomalies.
  - The first *BBS6* variant is c.748G > A (p.G250R), which was previously described in the homozygous state in two apparently unrelated Indian girls affected by retinitis pigmentosa, polydactyly, obesity, and developmental delay [4,5]. The minor allele frequency (MAF) reported in gnomAD is 0.001593%, with no homozygotes seen; the highest population frequency is South Asians (0.009799%). The variant results in a highly conserved amino acid’s nonconservative substitution (hydrophobic to basic). It is reported in the Human Gene Mutation Database (HGMD) (CM100875) and currently has one variant of uncertain significance (VUS) classification reported in ClinVar (649204). Overall, this *BBS6* variant pathogenicity classification is likely pathogenic.
  - The second *BBS6* variant is c.1643G > A (p.G548D), a nonconservative (hydrophobic to acidic) substitution of a highly conserved amino acid. This variant is not reported in the HGMD or ClinVar (although an adjacent variant, p. Leu549Pro, is reported as CM145492 in the HGMD). The MAF of this variant is 0.001593%, with the highest MAF in South Asians at 0.006540% (Exome Aggregation Consortium—ExAC). No prior reports of this variant were identified in the literature. Overall, this *BBS6* variant pathogenicity classification is a variant of uncertain significance.
2. Deafness, autosomal recessive 16 (OMIM:603720) due to a homozygous pathogenic variant in stereocilin (*STRC*). The diagnosis of autosomal recessive deafness was consistent with her clinical features of progressive moderate–severe sensorineural hearing loss requiring bilateral hearing aids. Together with her BBS disease, this pathogenic variant may account for her progressive moderate–severe bilateral SNHL [6].
  - The *STRC* c.4701 + 1G > A variant alters a consensus donor splice site and is predicted to shift the splicing pattern, resulting in a premature termination codon and protein truncation. The MAF is 0.003891%, with the highest frequency in South Asians at 0.02613%; no homozygotes are reported in gnomAD. It affects the consensus splice donor site (at +1) and is predicted to abolish splicing, likely skipping the 24th coding exon. This exon skipping affects the only known transcript [NM_153700.2]. It has been classified as P/LP in ClinVar by two clinical laboratories (variant ID 165305) and reported in HGMD (CS1824593). It was reported in a compound heterozygous patient with mild-to-moderate bilateral SNHL with a conductive component [7]. Although the *STRC* gene is well known to have a pseudogene, the patient’s hearing loss is consistent with the disease [8]. Overall, this *STRC* variant pathogenicity classification is pathogenic.
3. Thyroid dyshormonogenesis 6 (OMIM:607200) due to a homozygous dual oxidase 2 (*DUOX2*) variant. The diagnosis of thyroid dyshormonogenesis is consistent with her clinical features of congenital hypothyroidism diagnosed in early infancy and requiring lifelong thyroid replacement therapy and imaging analysis demonstration of small pituitary gland size.
  - The *DUOX2* variant c.1709A > T (p.Q570L) leads to a nonconservative substitution of a highly conserved amino acid in the peroxidase-like domain and is reported in the HGMD (CM1313825). This is a known recurrent pathogenic variant in the South Asian congenital hypothyroidism population, with a MAF of 0.1308% and the highest population frequency in South Asians (0.9773%). It has been reported in homozygous and compound heterozygous patients [9,10]. Functional studies proved this variant to be associated with reduced activity of *DUOX2* [10].Overall, this *DUOX2* variant pathogenicity classification is pathogenic.
4. Congenital cardiac minor anomaly. The patient carries a novel homozygous variant in cardiac Troponin *T2*, *TNNT2*, a gene associated with multiple autosomal dominant cardiomyopathies (OMIM: 601494, 612422, 115195). However, our patient has no evidence of cardiomyopathy and had normal echocardiograms at ages 13 and 18. She has a congenital mitral valve anomaly: a cleft mitral valve with mild mitral valve regurgitation that has not been clinically significant or previously associated with *TNNT2* variants.
  - The c.94G > C (p.D32H) variant has not been reported in the HGMD or ClinVar. It has a MAF of 0.01074% (0 homozygotes), with the highest population frequency in South Asians (0.08492%). Interestingly, the variant is located in exon 5, which is present only in embryonic isoforms of cardiac TnT and spliced out in adult isoforms. This substitution introduces an essential/positive amino acid into a highly acidic/negative region, which may disrupt some of the critical functions of this region that is thought to be involved in embryonic cardiac contractility and tolerance to acidosis (reviewed in [11]). Overall, this *TNNT2* variant pathogenicity classification is a variant of uncertain significance.

In summary, we report an 18-year-old consanguineous Indian girl who was the product of an apparent first-degree union and presented with complex clinical anomalies and multisystem progressive disease now recognized from a range of clinical diagnostic and research-based genetic studies to result from pathogenic or likely pathogenic variants in at least three distinct nuclear genes, namely *BBS6*/*MKKS*, *STRC*, and *DUOX2*.

## 3. Discussion

An 18-year-old Indian girl who presented to the Genetics Clinic at the Children’s Hospital of Philadelphia since early childhood manifests a highly complex presentation now recognized as a result of at least three distinct genetic disorders. Based on her genome’s substantial regions of homozygosity, she is the likely product of an apparent first-degree union. Genetic testing, including targeted panel analysis, exome sequencing on a clinical basis, and a research-based review of her raw clinical exome data, led to identifying at least three causal gene disorders to explain distinct aspects of her complex phenotype. All variants are found at the highest frequency in the South Asian population, consistent with the proband’s known biological ancestry. Surprisingly, however, her Bardet–Biedl syndrome appears to have resulted from compound heterozygous variants in *BBS6*, as is consistent with their high South Asian population frequency, but would not be necessarily related to consanguinity. A homozygous novel variant of uncertain significance was also identified in *TNNT2* that is of unclear relation to her mitral valve cleft with mild regurgitation. She does not have evidence of cardiomyopathy typically seen with pathogenic variants in *TNNT2*. It remains possible that she harbors yet-unrecognized pathogenic variants that contribute to some of her other clinical problems that are not satisfactorily explained by these known disorders, such as schizophrenia and lymphatic issues.

The first clinical diagnosis in this patient explained by genetic testing was Bardet–Biedel syndrome. The patient carried two *BBS6*/*MKKS* variants likely causative for Bardet–Biedl syndrome 6. However, we cannot rule out a small copy number variant undetectable by the SNP array, and her biological parents were unavailable to confirm the phasing. Our proband’s clinical manifestations typical of *BBS6* include progressive truncal obesity, retinal dystrophy, ataxia, four-extremity postaxial polydactyly, learning disabilities, intellectual disability, and behavioral problems. Of note, she did not have diabetes mellitus (present in 6% of *BBS6* cases) or renal and pelvic anomalies (present in 53–82% of the patients) [12,13].

The second clinical diagnosis in this patient, explained by genetic testing, was bilateral SNHL. Consistent with our patient’s early-childhood diagnosis of SNHL that was progressive to moderate–severe intensity and required bilateral hearing aids, pathogenic variants in *STRC* cause autosomal recessive nonprogressive or progressive childhood-onset sensorineural hearing loss usually characterized by a mild-to-moderate high-frequency sloping audiogram [6].

The third clinical diagnosis in this patient, explained by genetic testing, was congenital hypothyroidism with a small pituitary gland. Biallelic mutations in *DUOX2* cause congenital hypothyroidism, and the homozygous variant carried by this patient is recurrent in the South Asian population.

It is uncertain if the patient’s mild cardiac congenital anomaly of mitral valve cleft relates to any identified genetic etiology. Specifically, the patient carries a homozygous *TNNT2* VUS in a developmentally regulated exon expressed during embryogenesis. While conditions currently known to be associated with *TNNT2* are inherited in an autosomal dominant fashion and involve cardiomyopathy that was not present in our case, variants in exon 5 are not well studied since they are intronic in the adult isoforms (NM_001001430.2:c.68-1592). Alternatively, the mild congenital heart anomaly may be a feature of her *BBS6*-related Bardet–Biedel syndrome. Unfortunately, the parental genotypes and phenotypes are available.

This case highlights the need to carefully evaluate multiple independent genetic etiologies for complex phenotypes, particularly in the case of consanguinity, rather than presuming that unexplained features are expansions of known gene disorders. Also, it provides a strong example of the utility of performing research-based analysis of clinical exomes in unrevealing evaluation for clinical features not explained by known diagnoses.

## Figures and Tables

**Table 1:** Molecular findings associated with disease phenotypes in a consanguineous Indian patient with a complex clinical presentation.

Condition (OMIM # N)	Gene (Transcript)	Nucleotide and Protein Change	Amino Acid Change	MAP (# N Homozygotes)	Conservation	Variant Classification
BARDET–BIEDL SYNDROME 6	*BBS6* (NM_018848.3)	c.748G > A p.G250R	Hydrophobic to basic	0.001593% 0 homozygotes	Highly conserved	Likely pathogenic
		c.1643G > A p.G548D	Hydrophobic to acidic	0.001593% (0 homozygotes)	Highly conserved	VUS
DEAFNESS, AUTOSOMAL RECESSIVE 16	*STRC* (NM_153700.2)	homozygous c.4701 + 1G > A	Splice variant	0.003891% (0 homozygotes)	Consensus splice donor sequence	Pathogenic
THYROID DYSHORMONO-GENESIS 6	*DUOX2* (NM_014080.4)	homozygous c.1709A > T p.Q570L	Hydrophilic to hydrophobic	0.1308% (9 homozygotes)	Conserved	Pathogenic
Cleft mitral valve with mild regurgitation *	*TNNT2* (NM_000364.2)	homozygous c.94G > C p.D32H	Acidic to basic	0.0107% (0 homozygotes)	Conserved	VUS (known gene for cardiomyopathy)

Notes. VUS a variant of uncertain significance. Minor allele frequency (MAF) and homozygote numbers were obtained from gnomAD 2.1.1. Conservation score determined from Alamut. Variant pathogenicity classification was determined according to ACMG criteria. Segregation analysis was not possible in this case due to the proband being adopted with no contact with or information on birth parents. # N: number. * These cardiological findings were not reported in association with variants in this gene linked to cardiac function.

## Data Availability

The authors confirm that the data supporting the 158 findings of this study are available upon reasonable request.
